# Event and Comment

**Published:** 1908-10-15

**Authors:** 


### DENTAL REGISTER.

**Vol. LXII. October 15, 1908. No. 10.**

### EVENT AND COMMENT.

### New Work for Dental Societies.

There has been little or nothing new added to the work of our dental society meetings since they were first organized fifty years ago. The Illinois State Society is about to inaugurate a scheme which is new and somewhat promising. Its object is to increase the literary output by a systematic educational propaganda. It takes up the oft repeated phrase that "the dentist is not a reader of his profession's literature," and proposes to change it so far as Illinois dentists are concerned. The scheme seems somewhat elaborate and too complicated to become practically permanent. The idea is to take four or five dental journals to start with and establish library centers all over the State where these books can be consulted by any one who desires, in writing a paper for a dental society for instance, to know where he can obtain access to all articles that may have been printed in these four or five dental journals during the past five years. These books will be read by a committee and every article fully indexed under specific headings, which will be printed on cards, which any one may have on application, for information as to where such articles as he may desire are in print. He will then go to these small libraries and consult the books he needs. Some five or six of these libraries are to be established in Chicago, and every center of professional activity will have one of these small libraries containing files of the five or six journals for the past five years.

The scheme is a good one and if it can be carried out as contemplated, there is no question but it will prove of incalculable benefit to many dentists who would like to contribute to their profession's literature, but simply don't know how. We see several good sized loop holes where the plan is liable to leak or fail to accomplish the high hopes of its authors; but we know the men that are back of it, and we have confidence in their tremendous efforts, which we trust will be sufficient to overcome lack of interest or knowledge which are the things to-day that prevent our rapid development as a scientific profession. We shall watch the progress of the work with great interest, because of its novelty and because we believe it will serve a good purpose in calling to the attention of many practitioners the value of our present literature. We have a considerable number of good journals, and for a five dollar bill—an amount which every respectable practitioner should possess—he can buy five journals of his own selection, which if he will carefully read, will stimulate him to not only a holy desire to read, but also to contribute from his own pen. If four practitioners living in a town or community would each subscribe for five dollars worth of journals, they could have a periodical library containing all the journals printed in the United States. If the fifth and sixth men would spend a like amount on new text books a respectable library of such works could soon be accumulated. A town containing ten dentists ought to have a complete library, museum and research laboratory. Wouldn't such a plan of reorganization be a grand work for every state society?

